# Suppression of Cardiogenic Edema with Sodium–Glucose Cotransporter-2 Inhibitors in Heart Failure with Reduced Ejection Fraction: Mechanisms and Insights from Pre-Clinical Studies

**DOI:** 10.3390/biomedicines10082016

**Published:** 2022-08-19

**Authors:** Ryan D. Sullivan, Mariana E. McCune, Michelle Hernandez, Guy L. Reed, Inna P. Gladysheva

**Affiliations:** Department of Medicine, University of Arizona College of Medicine–Phoenix, Phoenix, AZ 85004, USA

**Keywords:** edema, HFrEF, dilated cardiomyopathy, excessive extracellular fluid, fluid management, endothelial dysfunction, inflammation, cardiac remodeling

## Abstract

In heart failure with reduced ejection fraction (HFrEF), cardiogenic edema develops from impaired cardiac function, pathological remodeling, chronic inflammation, endothelial dysfunction, neurohormonal activation, and altered nitric oxide-related pathways. Pre-clinical HFrEF studies have shown that treatment with sodium–glucose cotransporter-2 inhibitors (SGLT-2i) stimulates natriuretic and osmotic/diuretic effects, improves overall cardiac function, attenuates maladaptive cardiac remodeling, and reduces chronic inflammation, oxidative stress, and endothelial dysfunction. Here, we review the mechanisms and effects of SGLT-2i therapy on cardiogenic edema in various models of HFrEF. Overall, the data presented suggest a high translational importance of these studies, and pre-clinical studies show that SGLT-2i therapy has a marked effect on suppressing the progression of HFrEF through multiple mechanisms, including those that affect the development of cardiogenic edema.

## 1. Introduction

Heart failure (HF) affects about 64.3 million people worldwide [1], including an estimated 6.2 million in the United States [2]. The prevalence of symptomatic HF is expected to increase 46% by 2030, in comparison to 2012 [3]. HF with reduced ejection fraction (HFrEF) is characterized by progressive heart enlargement and declining contraction that leads to clinical symptoms (breathlessness, fatigue, swelling, etc.) from pathological fluid and sodium retention (edema) [4,5]. Sodium retention and the dysregulation of neurohumoral systems, including the sympathetic nervous system, renin–angiotensin–aldosterone system (RAAS), and the natriuretic peptide (NP) system, lead to excessive extracellular fluid accumulation in the interstitial space or edema (pulmonary, pleural effusion, ascites and/or gross/systemic peripheral fluid retention), which are hallmarks of symptomatic HF [5,6,7,8,9,10,11,12]. Symptomatic HF adversely impacts quality of life, is the primary cause of patient hospitalization, and is associated with premature mortality [13,14,15,16,17,18,19,20]. The prognostic role of edema is confirmed by clinical trials and post hoc analysis [13,19,21,22,23]. Maintaining physiologically relative fluid homeostasis is one of the primary goals of HF management [17,24].

During the last decade, significant progress has been made in the management of HFrEF with clearly defined, guideline-directed therapies [5,25,26,27,28,29,30,31]. Pharmaco-therapeutic interventions for HFrEF include modulating the neurohumoral response by targeting RAAS with angiotensin-converting enzyme inhibitors and angiotensin II (Ang II) receptor blockers; the sympathetic nervous system with beta-blockers; the mineralocorticoid system with mineralocorticoid receptor blockers (MRBs); and both RAAS and NP systems with combined Ang II receptor–neprilysin inhibitors (ARNI). However, better pharmacological interventions for reversing or preventing cardiogenic edema are needed. Early treatment at the pre-clinical stages may prevent HF progression to symptomatic stages with or without decompensation and improve outcomes. Most recently, the US Food and Drug Administration approved a new class of drug for the management of HF with reduced, mid-range or preserved ejection fraction called sodium–glucose cotransporter-2 inhibitors (SGLT-2i, originally developed as glucose-lowering agents), including canagliflozin, dapagliflozin, empagliflozin, and ertugliflozin [32,33]. In patients with HFrEF, SGLT-2i-based therapies enhance natriuresis/diuresis, modulate neurohumoral activation, improve cardiac and renal functions, functional status, duration, and quality of life [34]. These agents reduce HF-related hospitalization rates that are due to the aggravation of HF signs and symptoms caused by edema, independent from the reduction in glycemic level and the co-administration of guideline-directed HF therapy [34].

In HF, the retention of sodium and water by the kidneys leads to an expansion of free fluid in the interstitial compartments or edema. The expansion of the interstitial fluid volume is aggravated by vascular leakage caused by inflammation and endothelial dysfunction [35,36] and is associated with impaired extra fluid removal from the interstitial to intravascular space, which is in part caused by pathological alteration in the capillary dynamics and lymphatic system [24,37,38,39]. Fluid accumulation in the lungs manifests as cardiogenic pulmonary edema, while peripheral interstitial fluid overload manifests as soft tissue or third space edema. In contrast to classical diuretics, which affect blood plasma volume, SGLT-2i efficiently lowers edema by reducing pathological HF-related sodium retention and intestinal fluid volume [34,40,41,42,43]. Treatment with SGLT-2i also reduces the plasma level of N-terminal pro B-type natriuretic peptide (NT-pro-BNP) as an indicator of HF-associated reduced edema [44]. SGLT-2i receptor SGLT-2 is not expressed by cardiac tissue [45], excluding direct action of this class of drug on the heart. Therefore, the molecular and pathophysiological mechanisms of action of SGLT-2i in HFrEF remain inconclusive, although many intriguing hypotheses and mechanisms of action have been proposed [46,47,48,49].

This review aims to summarize the current evidence from pre-clinical translational studies, providing mechanistic experimental support for SGLT-2i action targeting the normalization of sodium–water homeostasis and the attenuation or prevention of edema signs and symptoms in HFrEF.

## 2. Contribution of Neurohumoral Activation, Cardiorenal Dysfunction and Cardiac Remodeling to Edema in HFrEF

Regardless of the etiology causing ventricular dysfunction, the ensuing compensatory mechanisms that occur in response to decreased cardiac output will be the same. Decreased cardiac output leads to a reduction in the intra-arterial effective circulating volume [24]. Subsequent sympathetic nervous system activation leads to an increase in heart rate and peripheral vasoconstriction. Concurrent RAAS activation and decreased renal perfusion lead to a reduction in sodium and water excretion from the kidneys [26,50]. Increased adrenergic tone and (Ang II)-induced vasoconstriction cause cardiac pressure overload. Increased intra-cardiac filling pressure leads to a backup of fluid in the pulmonary vasculature and the development of pulmonary congestion symptoms [9]. Increased water and sodium retention leads to intravascular blood volume expansion as well as interstitial fluid accumulation (extracellular water). Increased blood volume leads to increased central filling pressures and contributes to the development of peripheral congestion symptoms [9,24]. Edema directly increases pre-load as a feedback mechanism, contributing to left ventricular wall stress, cardiac remodeling and overall further decline in cardiac function [17].

During the asymptomatic stage of ventricular dysfunction (at risk for HF or at pre-HF stage [5]), the effects of elevated intra-cardiac filling pressures are mitigated by structural ventricular remodeling and the compensatory interdependent activation of neurohumoral systems. Thus, the early activation of the sympathetic nervous system and RAAS initially may help to maintain cardiac output and systemic perfusion. This compensatory response prevents the development of clinically evident signs or symptoms associated with the dysregulation of sodium–water homeostasis. As systolic dysfunction progresses, increased volume within the ventricles leads to an alteration in ventricular architecture, including myocyte hypertrophy/dilation, myocyte apoptosis, myofibroblast proliferation, and interstitial fibrosis [51]. Decreased forward flow as a result of decreased contractility leads to pathological neurohumoral activation, and the increased production of pro-atrial (ANP) and pro-B-type (BNP) natriuretic peptides by atria and ventricles as a compensatory mechanism. However, as HFrEF progresses, the NP system becomes impaired [52,53,54] and unbalanced persistent neurohumoral activation eventually becomes maladaptive and contributes to extracellular fluid retention or edema via vascular exchange to the interstitial space [26,41,50,55,56,57].

## 3. Evidence Supporting Edema Attenuation by SGLT-2i in Pre-Clinical HF Models

SGLT-2i inhibits renal sodium reabsorption and increases the urinary excretion of sodium, which may attenuate symptomatic HFrEF by reducing pulmonary and systemic edema and/or preventing edema formation [34]. Pre-clinical studies in animal models of HFrEF have shown that, in addition to natriuretic and osmotic/diuretic effects, treatment with SGLT-2i positively modulates edema-related plasma biomarkers and physiological outcomes contributing to edema modulation. Thus, SGLT-2i improves cardiac output and attenuates maladaptive cardiac remodeling, chronic inflammation, oxidative stress, and endothelial dysfunction (ED) by restoring the activity of nitric oxide (NO) within the vascular endothelium. The ability of SGLT-2i to attenuate inflammation and ED strongly suggests that SGLT-2i may help prevent fluid leakage from the vascular compartment to the interstitial space and prevent edema development. Despite the fact that the direct action of SGLT-2i on cardiac tissue is unlikely since SGLT-2 protein is not expressed in the heart, the combined impact of anti-inflammatory, anti-oxidative stress, and anti-ED effects of SGLT-2i may improve ventricular and global cardiac output, reduce fibrosis, suppress edema, and overall, attenuate HF progression.

### 3.1. Impact of SGLT-2i on Neurohumoral Activation toward Edema Restraining in HFrEF

SGLT-2i might retard HF-associated chronic activation of the sympathetic nervous system and RAAS, which stimulates salt and water retention by the kidneys and crucially contributes to pulmonary and systemic edema formation [26,58,59].

Experimental data related to the association between SGLT-2i treatment and RAAS modulation are limited to translational models of type 2 diabetes mellitus [60]. Overstimulation of the sympathetic nervous system in HFrEF is associated with elevated levels of norepinephrine in circulation [59]. Neurohormonal activation was attenuated in a group of non-diabetic pigs with post-MI HFrEF treated with empagliflozin, as demonstrated by reduced plasma levels of norepinephrine catabolites in comparison with the control experimental group [61]. Persistent sympathetic activation causes tachycardia [62]. In contrast to diuretics that elevate resting heart rate, SGLT-2i treatment is not associated with heart rate elevation [49] and may even reduce it [63], therefore suppressing pathologically elevated sympathetic activity. A trial in patients with HFrEF is warranted to align this mechanism with SGLT-2i outcomes in the clinic.

### 3.2. SGLT-2i Positively Affect Natriuretic Peptide System, Diuresis/Natriuresis, HF Signs, and Edema-Associated HF Plasma Biomarkers

The natriuretic peptide system plays a central role in natriuresis, diuresis, and vasodilatation, and balances the outcomes of the sympathetic nervous system and RAAS; however, in symptomatic HFrEF, its physiologic activity is impaired [52,54,64,65]. The associations between plasma BNP/NT-pro-BNP levels and edema were incorporated into the Universal Definition of Heart Failure [5]. In pre-clinical models of HFrEF, elevated plasma and cardiac pro-BNP/BNP (and pro-ANP/NT-pro-ANP) levels were strongly associated with extracellular fluid accumulation and clinically relevant edema manifestation as pleural effusion and pulmonary edema [53,56,66,67,68,69].

In a spontaneous hypertensive rat (SHR) HF model, treatment with empagliflozin reduced expression levels of ANP/BNP and tumor necrosis factor alpha (TNFα) in the ventricular tissue that were upregulated by HF [70]. In a zebrafish model of HF (induced by aristolochic acid), treatment with empagliflozin (0.1%, 10 µg) dampened the expression of ANP/BNP and downregulated related signaling pathways [66]. In an obese rat model of spontaneous hypertensive HF, empagliflozin treatment (925 mg/kg body weight by oral gavage for 6 months) decreased hepatic congestion [71]. In a post-myocardial infarction (MI) rat model of HFrEF, empagliflozin treatment increased urinary sodium excretion associated with a substantial reduction in body weight [72]. Utilizing a TAC-induced mouse HFrEF model, the beneficial effects of empagliflozin treatment on cardiac function were not dependent on natriuresis since the diuretic effect of SGLT-2i was not associated with any significant changes in electrolyte balance (blood or urine Na^+^ and K^+^ concentrations). ANP/BNP levels were not provided in this study [73].

### 3.3. SGLT-2i May Depress Edema by Improving Cardiac Function

Left ventricular function is an important indicator of HFrEF progression [1]. Reduced cardiac output stimulates chronic activation of the sympathetic nervous system and RAAS and promotes edema. The impact of SGLT-2i on systolic and diastolic function has been evaluated in rat, mouse and pig translational models of HFrEF. Several studies have investigated cardiac function outcomes in post-MI HFrEF models of left anterior descending (LAD) coronary artery ligation. In an ischemic reperfusion (IR) model of pre-diabetic obese insulin-resistant Wistar rats on a high-fat diet, dapagliflozin improved LVEF [74]. In a post-MI HFrEF model: (1) empagliflozin treatment increased LVEF [72], improved contractility, stroke volume, and end-systolic blood pressure despite diuresis, and improved diastolic function (reduction in LV end-diastolic pressure) [75]; canagliflozin IR model treatment alleviated left ventricular (LV) systolic and diastolic dysfunction, which may be explained by the increased phosphorylation of adenosine monophosphate-activated protein kinase, eNOS, and subsequent vasodilation [76]; (2) in non-diabetic mice, dapagliflozin treatment improved LV systolic function and LV mass [77]. In an MI porcine model, empagliflozin treatment improved LV systolic function [61] and ameliorated diastolic dysfunction [78]. In a model of cardiomyopathy induced by Ang II infusion in diabetic mice, dapagliflozin treatment increased LV fractional shortening [79]. In a genetic rat model of HFrEF (inducible diabetes and hypertensive HF), treatment with empagliflozin increased EF [80]. In a mouse model of doxorubicin (DOX)-induced cardiomyopathy, treatment with empagliflozin ameliorated LV dysfunction [81]. In a mouse model of cardiac pressure overload (by transverse aortic constriction (TAC)), the treatment of empagliflozin attenuated LV systolic and diastolic dysfunction, perhaps by increasing glucose and fatty acid oxidation [82]. Treatment with empagliflozin blunted the decline in cardiac function in a mouse model of TAC-induced HFrEF [73]. In a hypertensive HF model (spontaneous hypertensive rats fed a high fat diet for 32 weeks), treatment with empagliflozin normalized end-systolic and end-diastolic volume, but LV ejection fraction was not significantly improved [70]. Through the various species and models presented, SGLT-2i—at a minimum—maintains, but in most cases shows an overwhelming objective improvement in, cardiac function. Overall outcomes of SGLT-2i in translational models of HFrEF are summarized in Table 1.

### 3.4. SGLT-2i May Block Edema by Reducing Cardiac Remodeling

Pathological cardiac remodeling contributes to the development of edema and the progression of HF [5,51]. Beneficial effects of SGLT-2i treatment were demonstrated on inducible rat and mouse models of HFrEF (Table 1). In a hypertensive HF model (spontaneous hypertensive rats on a high-fat diet for 32 weeks), the treatment of empagliflozin significantly attenuated cardiac fibrosis in atrial and ventricular tissues [70]. Empagliflozin treatment attenuated adverse LV remodeling in post-MI HFrEF in pigs [61] and reduced interstitial cardiac fibrosis in pigs HFrEF induced by 2 h balloon occlusion of the proximal left anterior descending artery [78], which was associated with reduced extracellular volume [78]. In a rat post-MI model of HFrEF, empagliflozin treatment attenuated cardiomyocyte hypertrophy and fibrosis [72], but did not show any improvement in interstitial fibrosis or cardiomyocyte hypertrophy in another study [75]. In a mouse post-MI HFrEF model: empagliflozin treatment improved cardiac remodeling by the inhibition of apoptosis, alleviated oxidative stress, restored mitochondrial membrane potential, and activated AMPK signaling [83]; dapagliflozin treatment inhibited cardiac apoptosis and reduced LV mass, the expression of cardiac collagen 1/3, atrial natriuretic peptide (ANP), B-type natriuretic peptide (BNP), and transforming growth factor-β1 (TGF-β1) transcripts of cardiac fibrosis histological staining [77]. Treatment with empagliflozin (10 mg/kg/day) administered 3 weeks before MI improved cardiac remodeling and ameliorated fibrosis and hypertrophy post-MI in both diabetic and non-diabetic rats. This is possibly due to the increase in the myocardial expression of cardiac guanosine-5′-triphosphate enzyme cyclohydrase 1 (cGCH1), which activates neuronal nitric oxide synthase (nNOS) and endothelial nitric oxide synthase (eNOS) and inhibits inducible nitric oxide synthase (iNOS) [84]. In a mouse model of DOX-induced cardiomyopathy, treatment with empagliflozin (dosage not provided) lowered myocardial fibrosis [81]. In a rat model of cardiomyopathy (salt-sensitive hypertensive rats fed a high-salt/high-fat diet) treatment with tofogliflozin (0.005% for 9 weeks) reduced cardiomyocyte hypertrophy, perivascular fibrosis and associated fibrosis genes (ANP, BNP and interleukin 6) [87]. In an Ang II-induced model of cardiomyopathy in diabetic mice, dapagliflozin treatment attenuated fibrosis [70]. Overall, SGLT-2i reduce cardiac remodeling pathology, help to maintain a healthy cardiac tissue architecture, and prevent diastolic dysfunction, thus preventing HF progression and edema development.

### 3.5. Impact of SGLT-2i on Cardiorenal Function Leading to Edema Suppression

Cardiac and renal function are highly interdependent and modulate sodium and water retention. Increased sodium retention by the kidneys leads to intravascular blood volume expansion, as well as interstitial fluid accumulation (extracellular water). SGLT-2i may restrict excessive sodium and water retention in the interstitial space of the kidney parenchyma and reduce edema formation in HFrEF [88]. In a rat chronic kidney disease model caused by 5/6 subtotal nephrectomy and DOX-induced dilated cardiomyopathy, empagliflozin treatment (20 mg/kg/day for 60 days) was associated with a lower kidney injury score, decreased myocardial fibrosis, inhibited LV remodeling, and decreased BNP protein level in LV (an indicator of HF/pressure overload). This may be explained by empagliflozin treatment causing downregulated autophagy, apoptosis, reduced markers of oxidative stress (NADPH oxidase, NOX-1, NOX-2) in renal tubular cells, decreased markers of DNA damage (phosphorylated histone H2AX) and mitochondrial damage (cytosolic cytochrome C), and increasing indicators of mitochondrial integrity (mitochondrial cytochrome C) [89].

### 3.6. SGLT-2i Block Activation of Sodium–Hydrogen Exchangers in the Heart and Kidneys That Contribute to the Clinical Progression of HFrEF Associated with Edema

SGLT-2i may slow edema development and HF progression by blocking the activity of sodium–hydrogen exchangers (NHE) expressed in the myocardium (NHE-1 isoform) and in the proximal convoluted tubule of kidneys (NHE-3 isoform) [90], as well as the activity of the late component of the cardiac sodium channel current in cardiomyocytes [91]. NHE-1 regulates cardiomyocyte pH and volume. NHE-3 is responsible for the reabsorption of approximately 70% of filtered sodium [90]. In HFrEF, chronic neurohumoral activation stimulates the activation of cardiac NHE-1 and renal NHE-3, leading to enhanced sodium retention that contributes to the physiological and clinical progression of HFrEF associated with fluid retention (edema) and increased sodium influx and intracellular calcium linked to cardiac hypertrophy, cell injury and fibrosis [90,92,93].

Excess intracellular calcium within cardiac myocytes also increases arrhythmogenicity due to increased cytosolic calcium during the relaxation phase of the cardiac cycle. The inhibition of NHE-1 activity with cariporide in animal models of HF adequately restored sodium and calcium handling, caused the regression of ventricular hypertrophy, and improved several markers of electrophysiological remodeling such as reduced QT and QRS intervals [94]. SGLT-2i antagonizes the effects of NHE in the heart and kidneys. An in silico analysis of the mechanism of action of empagliflozin showed that it binds to and inhibits the downstream signaling effects of NHE activation. The NHE blockade prevents cardiomyocyte death by increasing the expression of apoptotic inhibitors in cardiomyocytes. These findings were validated by an in vivo HF rat model, which showed that treatment with empagliflozin led to an increased expression of anti-apoptotic proteins and slowed HF progression [95]. However, several studies do not support empagliflozin as a potent inhibitor of NHE-1 in the healthy heart [96,97].

### 3.7. SGLT-2i May Restrain Edema by Suppressing Chronic Inflammation and ROS

Chronic inflammation and oxidative stress promote maladaptive systolic dysfunction, cardiac remodeling, and pulmonary/systemic edema, and therefore are hallmarks of HFrEF pathophysiology [36,98,99,100]. Treatment with SGLT-2i decreased inflammation and/or the production of reactive oxygen species (ROS) in inducible and genetic rat/mouse models of HFrEF. Empagliflozin treatment attenuated oxidative stress [72], and dapagliflozin treatment lowered cardiac transcripts of inflammatory cytokines (TNFα, TGF-β1, Vcam-1, MCP-1, Icam-1, IL-6) [77] in mouse/rat MI models. Incardiac dysfunction mouse models induced by administering lipopolysaccharide (LPS, 5 mg/kg) co-administration of empagliflozin preserved cardiac function possibly by improved AMPK phosphorylation and ATP/ADP, as well as reduced cardiac iNOS, plasma TNFα, and creatine kinase MB (isoenzyme, found mostly in cardiac and some skeletal muscles) levels [86]. In a model of cardiomyopathy by Ang II infusion in diabetic mice, dapagliflozin decreased inflammation and ROS production [70]. In a genetic rat model of HFrEF (inducible diabetes and hypertensive HF), treatment with empagliflozin decreased the infiltration of macrophages [80]. In a mouse TAC-induced model of HFrEF, empagliflozin decreased the expression of markers of sterile cardiac inflammation, possibly by attenuating the activity of the neutrophil-to-lymphocyte ratio (NLR) family pyrin domain containing 3 (NLRP3) inflammasome; this occurred in the absence of changes to ketone bodies or cardiac ATP use [73]. SGLT-2i therapy clearly attenuates cardiac inflammation and ROS associated with HFrEF.

### 3.8. SGLT-2i May Prevent Vascular Leakage and Edema by Improvement of Endothelial Dysfunction

The endothelium is a semi-permeable barrier that plays a crucial role in tissue fluid hemostasis by the tight control of fluid exchange from the vascular compartment to the interstitial space [101]. The proper function of the vascular endothelium is vital for preventing the inadvertent extravasation of fluid into surrounding tissues or edema. Clinical and translational HF is associated with a significant impairment of endothelium-dependent vasodilation (endothelial dysfunction (ED)), which is mediated almost entirely by the excess formation of superoxide radicals and other oxidant species that interfere with the activation of nitric oxide (NO) and the bioavailability of cyclic guanosine monophosphate (cGMP) [35,69,102]. Thus, in chronic HF, increased oxidative stress and the dysregulation of NO pathways lead to coronary/peripheral arteries and/or lung ED, which contribute to HF decompensation (NYHA class IV, pulmonary edema) and are associated with hospitalization and heart transplantation [103,104,105].

In HFrEF, treatment with SGLT-2i may prevent vascular leakage leading to edema by the prevention or improvement of ED. The impact of SGLT-2i on attenuating ED by restoring the activity of NO within the vascular endothelium in pre-clinical and clinical studies has been comprehensively overviewed [106,107,108,109]. Several pre-clinical studies have demonstrated that one of the off-target effects of SGLT-2i may involve a reduction in ED [106,107]. These pre-clinical studies were predominantly conducted on models of diabetes, since ED is known to be a major mediator of diabetic vascular disease. In a porcine model of HFrEF induced by 2 h balloon occlusion of proximal LAD artery, treatment with empagliflozin increased the activity of eNOS and NO production and the bioavailability associated with the activation of the cGMP–PKG axis [78]. Ex vivo studies have demonstrated that SGTL-2i may directly induce vasodilation by several mechanisms, including the modulation of cell adhesion molecules, the attenuation of inflammation, and reduced oxidative stress [106]. Treatment with empagliflozin was associated with a significant improvement in endothelial-dependent vasodilation in streptozotocin (STZ)-induced type 1 diabetes mellitus (T1DM) rat models [107]. Empagliflozin has been found to improve the enzymatic activity of eNOS, a key enzyme in the production of the one of the most important mediators of vasodilation, NO, in STZ-induced diabetic rats [107]. Thus, SGLT-2i may exert its vasodilatory effects by restoring the activity of NO within the vascular endothelium. Another possible mechanism by which SGLT-2i carriy out their vasorelaxant effect is by inhibiting glucose-mediated membrane depolarization [110]. One study found that canagliflozin and phlorizin induced membrane hyperpolarization in pulmonary artery smooth muscle cells. This finding was attributed to the activation of potassium channels in the plasma membrane of these cells by NO [110]. Empagliflozin restored the beneficial effect of cardiac microvascular endothelial cells on cardiomyocyte function in a co-culture system of human cardiac microvascular endothelial cells with adult rat ventricular cardiomyocytes by reducing mitochondrial oxidative damage and, ROS accumulation, and increasing the bioavailability of endothelial NO [109]. Still, an investigation of SGLT-2i treatment outcomes on pre-clinical model(s) of DCM-HFrEF characterized by impaired NO-cGMP bioavailability and cardiac eNOS production [69] is warranted.

### 3.9. Alteration of Cardiac Metabolism and Energy Utilization by SGLT-2i Improves Cardiac Structure and Function, Which May Contribute to Edema Reduction

As noted in earlier sections, the effects of SGLT-2i on cardiac tissue are largely indirect. However, cardiac metabolism is directly altered with the inclusion of SGTL-2i therapy. The failing heart is characterized by altered cardiac muscle contraction, increased oxygen demand, and high cellular turnover, resulting in an increased metabolic demand to support the pathologic condition which can manifest symptomatically as cardiac cachexia and sarcopenia [56,111]. By lowering circulating glucose levels, SGLT-2i shifts the cardiac metabolism to function under a fasting-like state (catabolic), with energy provided by gluconeogenesis and ketogenesis [112]. The energy-deficient state increases cardiac autophagy (cellular cleanup) by activating the SIRT1/PGC-1α/FGF21 pathway, thus reducing local inflammation, lowering oxidative stress, and removing dysfunctional cells that may otherwise contribute to myocardial remodeling as a consequence of cellular necrosis [112]. Ketone utilization produces more ATP than glucose or other metabolites [113], thus improving the energy supply for HFrEF. The increased availability of cardiac energetics with SGLT-2i therapy has been shown to improve cardiac structure and function in mice with and without T2DM [114,115]. These improvements should reduce cardiogenic-associated edema in HFrEF models treated with SGLT-2i therapy, though additional studies are needed to specifically investigate this correlation.

## 4. Limitations

Pathological RAAS overactivation and the impairment of the NP system significantly contributes to sodium retention and fluid accumulation in the interstitial space leading to edema. However, pre-clinical studies targeting the impact of SGLT-2i on these systems and overall neurohumoral activation are currently lacking. In HFrEF clinical and pre-clinical studies, treatment with SGLT-2i leads to an overall improvement of left ventricular function and the attenuation of cardiac remodeling, which are essential promoters and indicators of edema development and HF progression. Still, the direct mechanisms responsible for such beneficial action of SGLT-2i remain unclear, since cardiac tissue lacks the SGLT-2i receptor SGLT-2.

One major issue surrounding clinical and pre-clinical cardiogenic edema is the ability to detect early changes in excess fluid during the transition from the pre-symptomatic to the symptomatic phase of HF. Current diagnostic imaging modalities include echocardiography, MRI, CT, and thoracic radiography, though they often require specialized training for image collection and analysis. Quantitative magnetic resonance (QMR) has been introduced as an objective and longitudinal method for diagnosing and monitoring systemic cardiogenic edema in animal models [56,67,69,116,117], which may offer a refined method for monitoring edema therapeutic response throughout all phases of HF.

The locomotive and anatomical differences between human and animal models (mice, rats, pigs) used should also be considered. Upright (bipedal) versus horizontal (quadrupedal) orientation may have translational differences for the effects of edema development, manifestation, and clearance. Known differences include: pedal edema (humans), which is less commonly observed in the forelimbs of animals exhibiting HF due to changes in subcutaneous space [118]; the alteration of fluid lines and pulmonary patterns in thorax imaging due to gravity-dependence [119]; and evolutionary changes in baroreceptor sites and function throughout mammalian species [120]. Additional considerations for managing hospitalized HF patient positioning (horizontal recumbent, supine) when undergoing treatment and care for cardiogenic edema should be examined, as unanesthetized HF animal models rarely adopt this orientation. Newer studies suggest proning edema patients, and thus placing them in a more animal-like posture [119,121] to improve outcomes.

SGLT-2i is recommended in the outpatient setting for all patients with stable HFrEF conditions [122]. As with most pharmacological treatments, side effects and limitations for use have been reported from clinical trials and medical practice with SGLT-2i [123,124,125]. The most common clinical side effects might include fatigue, nausea, increased drinking, sudden urge to urinate, dry mouth, female urinary tract infections, hypotension, changes in blood pH, and, more rarely, acute renal pathology, bone fractures, and lower limb amputations. As a result of study design and inability to communicate directly with the patient, unfortunately, most pre-clinical studies are not able to account for all potential side effects prior to advancing to human administration.

## 5. Conclusions

**Figure 1:**
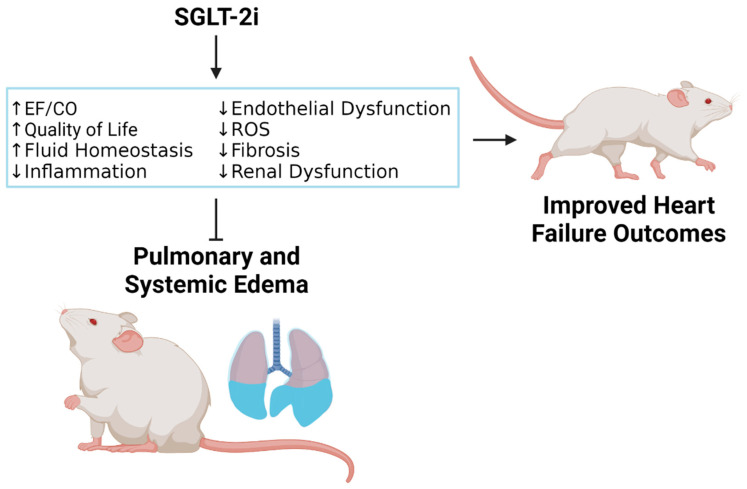
Mechanism by which sodium–glucose cotransporter 2 inhibitors (SGLT-2i) reduce pulmonary and systemic edema in HFrEF. Ejection fraction (EF); cardiac output (CO); reactive oxygen species (ROS). Created with BioRender.com on 18 July 2022.

Effective edema prevention and treatment are the primary goals of HF management and an unmet need to improve quality of life, reduce HF-related hospitalization rate, and prolong life. HFrEF pre-clinical studies have demonstrated that SGLT-2i treatment may attenuate edema formation through the stimulation of natriuretic and osmotic/diuretic effects, improvement in overall cardiac function, and the suppression of maladaptive cardiac remodeling, chronic inflammation, oxidative stress, and endothelial dysfunction (Figure 1). By repressing inflammation and endothelial dysfunction, SGLT-2i may prevent vascular leakage and edema development associated with extensive fluid accumulation in the interstitial space. In addition to its anti-inflammatory, anti-oxidative stress, and anti-fibrotic effects, SGLT-2i improves ventricular and global cardiac output, suppresses edema, and slows the rate of HF progression, locally in terms of cardiac function, and systemically at the kidneys, which appear to be the primary site of action.

## Figures and Tables

**Table 1:** Cardiac outcomes of treatments with SGLT-2i in pre-clinical models of HFrEF.

Preclinical Models (species)	Drug Dosage Duration	Cardiac Function	Cardiac Remodeling	Inflammation, ROS, ED
Hypertensive HF model (rat)	Empagliflozin 20 mg/kg/day 12 weeks [70]	Normalized end diastolic, end systolic volume. LVEF not significantly improved [70]	Reduced cardiac fibrosis [70]	
Genetic HFrEF model (rat)	Empagliflozin 10 mg/kg/day 4 weeks	Increased cardiac function and LVEF [80]		Decreased infiltration by macrophages [80]
Post-MI HFrEF model (rat; mouse, pig)	Empagliflozin 10mg orally 2 months [61]	Increased LV systolic volume [61]	Attenuated remodeling post-MI (lower LV, dilation, sphericity) [61]	
	20 mg/kg/day 6 weeks [75]	Improved contractility, stroke work, end-systolic blood pressure diastolic function [75]	No improvement in interstitial fibrosis or cardiomyocyte hypertrophy [75]	
	30 mg/kg/day 2 weeks [72]	Improved LVEF [72]	Attenuated cardiomyocyte hypertrophy, fibrosis [72]	Decreased inflammation alleviated oxidative stress [72,83, 84, 85];
	10 mg/day 2 months [78]	Improved diastolic function [78]	Ameliorated diastolic dysfunction [78]	Reduced extracellular volume [78] Increased eNOS activity and NO production and bioavailability associated with cGMP-PKG axis [78]
	10 mg/kg/day 2 weeks [81]		Improved cardiac remodeling [81]	
	Dapagliflozin 1 mg/kg/day 28 days [72, 74]	Improved LVEF [72, 74]		
	1.5 mg/kg/day 4 weeks [77]	Systolic function [77]	Inhibited cardiac apoptosis and reduced LV mass, cardiac collagen 1/3, ANP/BNP, TGF-β1 transcripts, cardiac fibrosis [77]	Lowered levels of inflammatory cytokines [77]
	Canagliflozin 3 µg/kg 5 mins [76, 82]	Alleviated LV systolic and diastolic dysfunction [76, 82]		
Ang II-induced cardiomyopathy (mouse)	Dapagliflozin 1.5 mg/kg/day 30 days [79]	Increased LV fractional shortening [79]		Decreased inflammation and ROS production [79]
DOX-induced cardiomyopathy (mouse)	Empagliflozin (not provided) [81]	Ameliorated LV function [81]	Lowered myocardial fibrosis [81]	
LPS-induced cardiomyopathy (mouse)	Empagliflozin 5 mg/kg [86]	Preserved cardiac function [86]		Reduced cardiac iNOS, plasma TNFα and creatine kinase MB [86]
TAC-induced HFrEF (mouse)	Empagliflozin 10 mg/kg/day 2 weeks post-surgery [73, 82]	Attenuated the decline in cardiac function [73, 82]	Attenuated LV remodeling [82]	Decreased expression of markers of cardiac inflammation [73]

Heart failure (HF); Left ventricular ejection fraction (LVEF); Heart failure with reduced ejection fraction (HFrEF); Left ventricular (LV); Angiotensin II (Ang II); Reactive oxygen species (ROS); Endothelial dysfunction (ED); Doxorubicin (DOX); lipopolysaccharides (LPS); Inducible nitric oxide synthase (iNOS); Tumor necrosis factor alpha (TNFα); Transverse aortic constriction (TAC).

## Data Availability

Not applicable.
